# KSHV RTA Abolishes NFκB Responsive Gene Expression during Lytic Reactivation by Targeting vFLIP for Degradation via the Proteasome

**DOI:** 10.1371/journal.pone.0091359

**Published:** 2014-03-10

**Authors:** Elana S. Ehrlich, Jennifer C. Chmura, John C. Smith, Nene N. Kalu, Gary S. Hayward

**Affiliations:** 1 Viral Oncology Program, The Sidney Kimmel Comprehensive Cancer Center, Johns Hopkins University School of Medicine, Baltimore, Maryland, United States of America; 2 Department of Biological Sciences, Towson University, Towson, Maryland, United States of America; University of Southern California Keck School of Medicine, United States of America

## Abstract

Kaposi's sarcoma herpesvirus (KSHV) is a gamma-2 herpesvirus present in all cases of Kaposi's sarcoma, primary effusion lymphoma (PEL), and some cases of multicentric Castleman's disease. Viral FLICE inhibitory protein (vFLIP) is a latently expressed gene that has been shown to be essential for survival of latently infected PEL cells by activating the NFκB pathway. Inhibitors of either vFLIP expression or the NFĸB pathway result in enhanced lytic reactivation and apoptosis. We have observed a decrease in vFLIP protein levels and of NFκB activation in the presence of the KSHV lytic switch protein RTA. Whereas vFLIP alone induced expression of the NFĸB responsive genes ICAM1 and TNFα, inclusion of RTA decreased vFLIP induced ICAM1 and TNFα expression in both co-transfected 293T cells and in doxycycline induced TREx BCBL1 cells. RTA expression resulted in proteasome dependent destabilization of vFLIP. Neither RTA ubiquitin E3 ligase domain mutants nor a dominant-negative RAUL mutant abrogated this effect, while RTA truncation mutants did, suggesting that RTA recruits a novel cellular ubiquitin E3 ligase to target vFLIP for proteasomal degradation, allowing for inhibition of NFĸB responsive gene expression early during lytic reactivation.

## Introduction

Kaposi's Sarcoma Herpesvirus (KSHV), also known as human herpesvirus 8 (HHV8), is the causative agent of Kaposi's Sarcoma (KS) and is associated with primary effusion lymphoma (PEL) and multicentric Castleman's disease (MCD). As with all herpesviruses, KSHV has two phases of its lifecycle, latent infection and lytic replication. During latency, only five genes are expressed, and the genome is maintained as a multicopy episome tethered to the chromatin by the Latency-Associated Nuclear Antigen (LANA) [1], [2]. Lytic replication is initiated via expression of the viral regulator of transcription activation (RTA) protein both directly in primary infection and as a lytic switch factor during reactivation [3].

Viral FLICE inhibitory protein (vFLIP) is a latently expressed protein that has homology to the cellular FLIPs which function as inhibitors to death receptor induced apoptosis. vFLIP has also been shown to activate NFκB by interacting with the IκB kinase complex (IKK) [4]–[7]. This occurs via activation of IKK followed by phosphorylation of IκB and subsequent translocation of NFκB into the nucleus [8], [9]. Knockdown of vFLIP by siRNA in PEL cell lines results in apoptosis, consistent with its reported anti-apoptotic activity [10]. Chemical inhibition of NFκB by Bay11–7082 in PEL cells promotes lytic reactivation while activation of NFκB inhibits lytic promoters [11], [12]. This data suggests that vFLIP induced NFκB activation is required for cell survival and maintenance of latent infection and must be negatively regulated in order for the virus to enter the lytic replication cycle.

Aside from being the major switch for reactivation of KSHV from latency, RTA has been assigned intrinsic E3 ubiquitin ligase activity [13]. RTA has been shown to specifically target IRF7 for proteasomal degradation as a mechanism to abrogate the interferon α/β response to viral infection [13]. In addition, RTA has been reported to degrade a number of known RTA repressors such as Hey1, LANA and NFκB (p65) [14], [15]. More recently RTA has been shown to also recruit and stabilize the cellular ubiquitin ligase RAUL in order to target both IRF3 and IRF7 for proteasomal degradation [16].

Here we provide evidence that RTA targets vFLIP for proteasomal degradation resulting in downregulation of NFκB responsive gene expression early during lytic reactivation. RTA expression inhibited vFLIP induced NFκB activation as well as vFLIP-mediated NFκB responsive gene expression. In particular, vFLIP induced ICAM1 and TNFα gene expression in 293T cells and this gene expression was abolished upon expression of RTA. We observed similar transient down-regulation of ICAM1 and TNFα in doxycycline-induced TREx BCBL1-RTA cells. Expression of RTA resulted in dose dependent destabilization of vFLIP and this effect was abrogated by treatment with proteasome inhibitors. RTA and RAUL ubiquitin ligase mutants did not affect RTA induced vFLIP downregulation, while several RTA truncation mutants displayed a moderate defect in vFLIP degradation and were unable to down-regulate vFLIP induced TNFα and ICAM1. Taken together this data suggests that RTA down-regulates vFLIP induced NFκB activation and associated gene expression through recruitment of another as of yet unidentified cellular ubiquitin ligase.

## Materials and Methods

### Cells and transfection

293T and Vero cells were maintained in DMEM supplemented with 10% FBS. TREx BCBL1-Rta cells (a generous gift from Dr. Jae Jung) were maintained in RPMI supplemented with 20% FBS and 200 µg/ml hygromycin B [23]. RTA expression was induced with 1 µg/ml doxycycline. All cells were grown at 5% CO_2_ at 37**°**C. Cells were transfected at 60–70% confluence using 1 mg/ml polyethyleneimine (PEI) linear, MW∼25,000 (Polysciences, Inc. Cat#23966) at a ratio of 1 ug plasmid DNA:3 ul PEI.

### Reagents and effector and reporter genes

The following expression plasmids were used in this study: pYNC989 (vFLIP-myc), pNF-kB-Luc (Stratagene), pGL4.70[hRluc] (Promega) and pcDNA3.1. pCMV-myc-RAUL-WT and the pCMV-myc-RAUL dominant negative mutant (C1051A) were provided by Cecile Pickart [17]. pSEW R01 (WT RTA), pSEW R03, pSEW R04, pSEW R06, pSEW R11 and RTA H145L have been described previously [13], [18]. V5 tagged RTA 11–149 was cloned into pcDNA3.1 using the following primers: F- 5′-ATCGGAATTCGTTATGGTTCGGCGGTCCTGTGTGGAAAG and R-5′-TATATCTAGATTTTCACGTAGAATCGAGACCGAGGAGAGGGTTAGGGATAG GCTTACCCAGGCATTTGGCCTTCATTTCAG. The following reagents were used in this study: MG132 (Boston Biochem), doxycycline (Sigma), hygromycin (Roche), and antibodies anti-cmyc (Millipore), anti-tubulin (Sigma), anti-β-actin (Sigma), rabbit anti-RTA and rabbit anti-RAUL (24, 25).)

### Luciferase reporter assay

NFĸB activation was quantified using the Dual Luciferase reporter assay system (Promega). Briefly cells were transfected with indicated plasmids plus the two reporter plasmids pNFĸB-Luc (Stratagene) and pGL4.70[hRluc]. Cell lysates were prepared according to the manufacturer's protocol. Luciferase was measured on a GloMax®-Multi Microplate Multimode Reader (Promega). Data were taken as a ratio of firefly/renilla luciferase.

### Immunoblot analysis

Cells were harvested by direct addition of 2.5× Laemmli Buffer followed by incubation at 100**°**C for 5 min. Lysates were run on a 10% SDS-PAGE or Any kD Mini-PROTEAN TGX Precast Gel (Biorad) and Tris-glycine running buffer. Proteins were transferred to a PVDF membrane using a semi-dry transfer system at 25 V for 20 min. Membranes were blocked in 5% non-fat dry milk in PBS for 1 hr followed by incubation with primary antibody overnight at 4**°**C and secondary antibody for 1 hr at room temperature. Proteins were visualized by addition of ECL substrate and detection of chemiluminescent signal on x-ray film.

### RNA extraction, reverse transcription, and real-time RT-PCR

RNA was extracted using the Promega SV Total RNA Extraction Kit. DNase-treated RNA was reverse transcribed using the RevertAid First Strand cDNA Synthesis Kit (Fermentas) according to manufacturer's instructions. The resulting cDNA was used for qPCR performed on an ABI Prism 7000 Sequence Detection System using Maxima SYBR Green/ROX qPCR Master Mix (Thermo Fisher Fermentas) as per manufacturer's specifications. vFLIP, ICAM1, TNFα, GAPDH, and Beta-actin were amplified using the following primers: B-actin F 5′-CAT GTA CGT TGC TAT CCA GGC-3′, R 5′-CTC CTT AAT GTC ACG CAC GAT-3′; GAPDH F 5′-AAT CCC ATC ACC ATC TTC CAG-3′, R 5′-AAA TGA GCC CCA GCC TTC-3′; ICAM1 F 5′-CAA TGT GCT ATT CAA ACT GCC C-3, R 5′-CAGCGTAGGGTAAGGTTCTTG-3′; TNFα F 5′-ACT TTG GAG TGA TCG GCC-3′, R 5′-GCT TGA GGG TTT GCT ACA AC-3′; vFLIP F 5′- GGATGCCCTAATGTCAATGC-3′, R 5′- GGCGATAGTGTTGGAGTGT-3′. Relative gene expression was calculated using the ΔΔCt method using GAPDH and β-actin housekeeping genes for normalization. Data was analyzed for statistical significance using a fixed-effects one-way ANOVA with alpha set at 0.05 using JMP (Version 10.0). Error bars represent the standard error. Human NFκB Signaling Pathway RT^2^Profiler PCR Array was obtained from SABiosciences (QIAGEN). Samples were prepared, run and analyzed according to the manufacturers instructions.

### Immunofluorescence assay

293T cells were grown and transfected in chamber slides as indicated. Cells were fixed with paraformaldehyde and permeablized with 0.3% Triton-X 100. Cells were stained with the indicated primary antibodies for 1 hr, followed by washing with PBS and staining with the appropriate fluorophore conjugated secondary antibody. Slides were washed and coverslips were mounted using Vector mounting media with DAPI. Cells were visualized using a Zeiss Axio Observer inverted fluorescence microscope.

## Results

### RTA inhibits vFLIP induced NFκB activation

**Figure 1:**
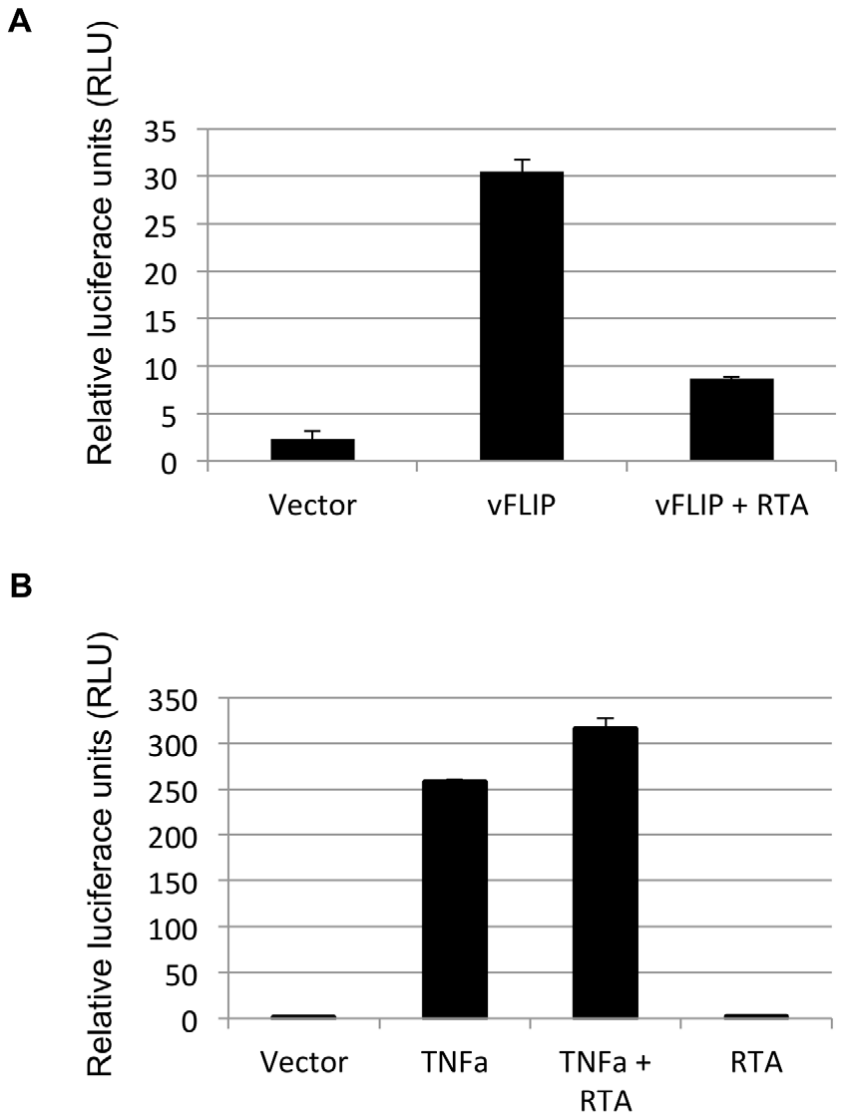
RTA inhibits vFLIP-induced NFκB activation. A. RTA expression inhibits vFLIP induced NFκB activation. 293T cells were transfected with vFLIP and RTA where indicated along with a luciferase reporter under control of an enhancer with five NFκB binding sites and a Renilla control plasmid. NFκB activity was detected as a ratio of Luciferase:Renilla using a Promega luminometer. B. RTA has no effect on TNFα induced NFκB activation. 293T cells were transfected with RTA and/or vFLIP along with a luciferase reporter under the control of an enhancer with five NFκB binding sites and a Renilla control plasmid. Cells were treated with 5 ng TNFα for 16 hrs where indicated. NFκB activity was detected as a ratio of Luciferase:Renilla activity using a Promega luminometer.

It is well established that vFLIP induces NFĸB activation [4]–[7], [9] and that activation of NFĸB is required for maintenance of latency [11], [12]. These data suggest that NFĸB associated gene expression might need to be down-regulated for the virus to enter the lytic cycle of replication. To determine whether RTA has an effect on NFĸB signaling, 293T cells were transfected with vFLIP and/or RTA and NFκB activity was assessed using a luciferase reporter containing five NFκB response elements. vFLIP expression induced a 15-fold NFκB activation as expected, however co-transfection with RTA reduced the activation three-fold (Fig. 1a). The role of RTA in blocking vFLIP induced NFĸB activation could be explained by one or more of the following mechanisms: RTA induced degradation of vFLIP or RTA-induced down-regulation of components of the NFĸB pathway. To evaluate whether RTA was targeting components of the NFκB pathway, we assessed the effect of RTA on TNFα induced NFκB activation. While co-transfection with RTA consistently resulted in a dose dependent 2–3 fold decrease in vFLIP induced NFκB activation, RTA had no significant effect on NFκB activation stimulated by TNFα, suggesting that RTA was targeting an aspect of vFLIP-induced NFĸB activation, rather than a component of the signal transduction pathway (Fig. 1b). We were also unable to find any evidence that RTA down-regulated either LANA nor NFĸB protein levels or those of components of the IKK pathway in the absence of vFLIP (data not shown). This data suggests that RTA expression counteracts latency by inhibiting vFLIP-induced NFκB activation by targeting vFLIP functions.

### RTA expression induces vFLIP degradation via the proteasome

**Figure 2:**
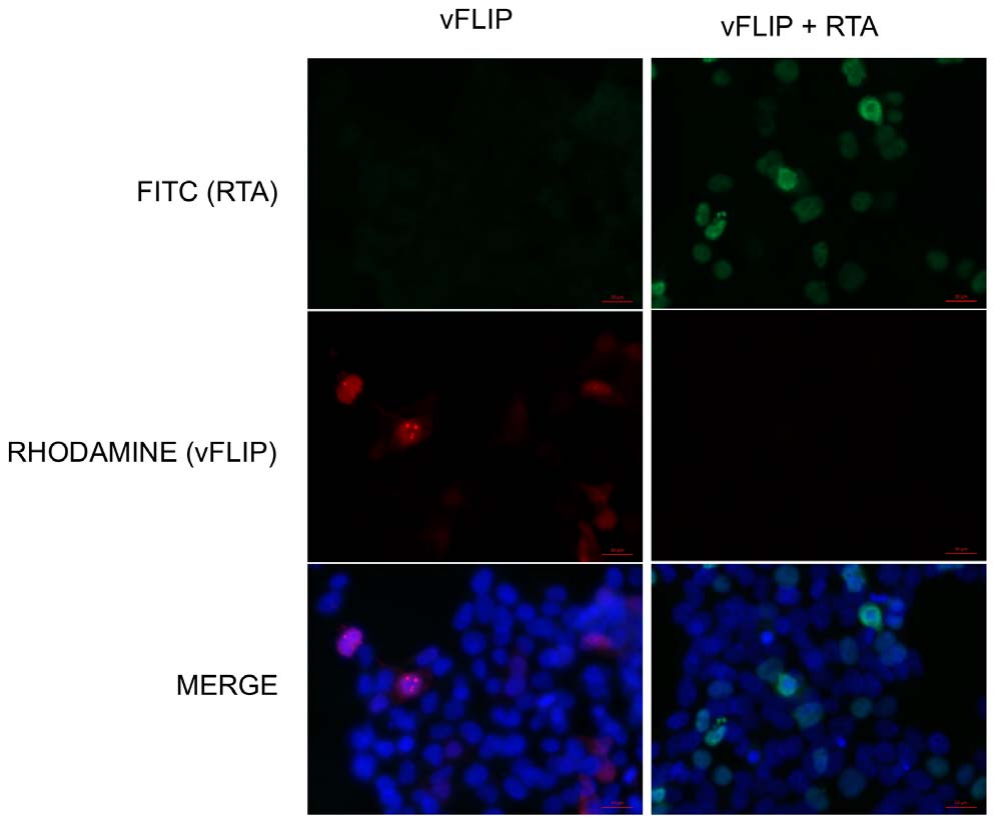
vFLIP is not visible in cells expressing RTA. vFLIP expression is decreased in 293T cells cotransfected with RTA. 293T cells were transfected with myc tagged vFLIP and RTA where indicated. vFLIP was detected with anti-myc antibody (Millipore) followed by a rhodamine conjugated secondary antibody (Jackson ImmunoResearch). RTA was detected with our own antibody against RTA followed by a FITC conjugated secondary antibody. Nuclei were visualized using a DAPI containing mounting media.

**Figure 3:**
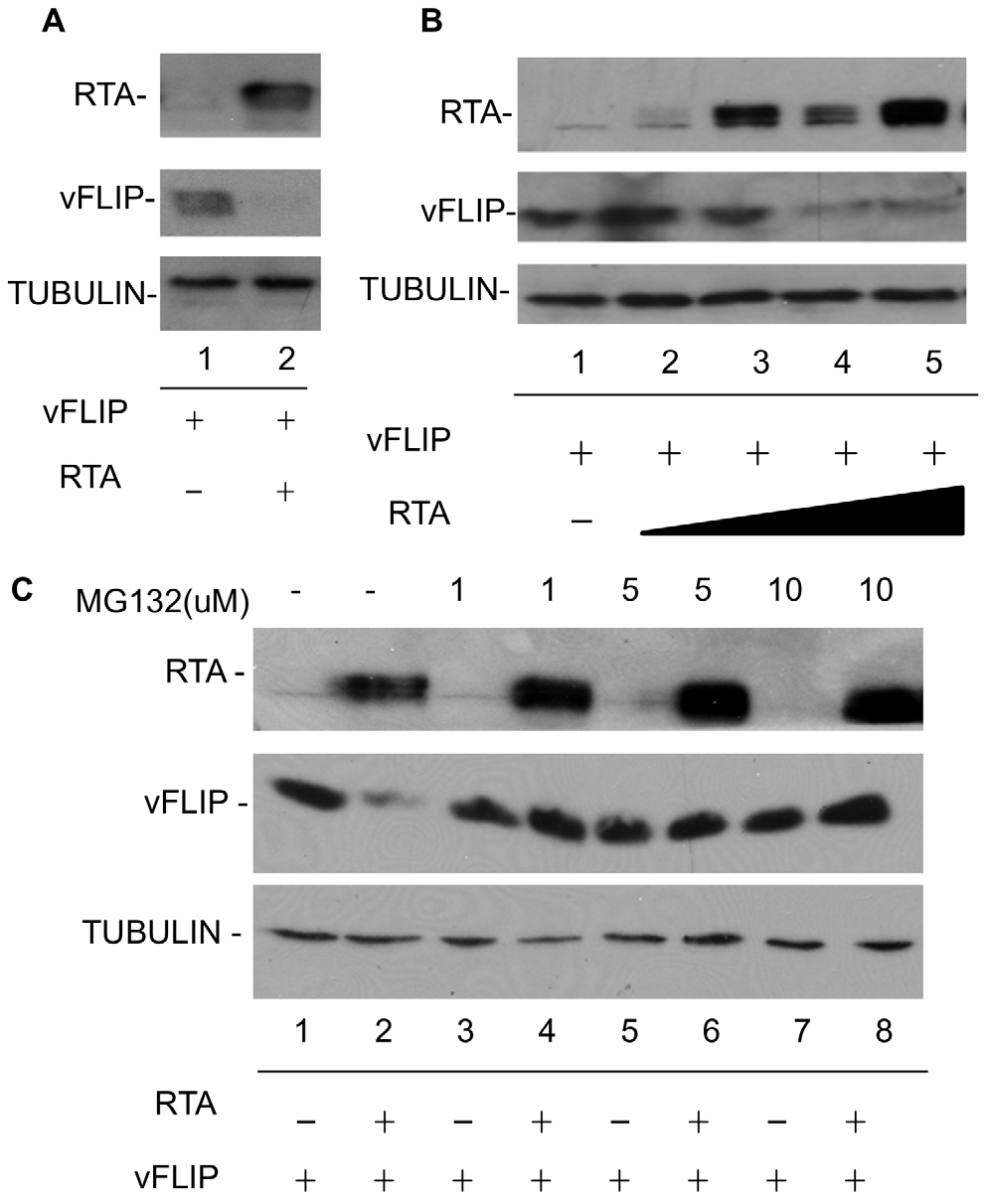
RTA expression alters vFLIP expression. A. vFLIP protein is decreased in the presence of RTA. 293T cells were transfected with myc-vFLIP, RTA, or empty vector where indicated. 48 hrs post-transfection, cells were harvested and analyzed by SDS-PAGE followed by western blot with antibodies against myc(vFLIP), RTA, and tubulin. B. RTA induced degradation of vFLIP is dose dependent. 293T cells were transfected with myc-vFLIP and increasing levels of RTA or empty vector control where indicated. 48 hrs post transfection, cells were harvested and analyzed by SDS-PAGE followed by western blot with antibodies against myc(vFLIP), RTA, and tubulin. C. RTA induces proteasomal degradation of vFLIP. 293T cells were transfected with myc-vFLIP and RTA or empty vector control where indicated. The proteasome inhibitor MG132 was added 12 hrs post transfection at the indicated concentrations. DMSO was added in the absence of MG132 in lanes 1 and 2. 48 hrs post transfection, cells were harvested and analyzed by SDS-PAGE followed by western blot with antibodies against myc(vFLIP), RTA, and tubulin.

RTA has previously been shown to participate in protein degradation either through its intrinsic ubiquitin ligase activity or through recruitment of the cellular ubiquitin E3-ligase RAUL [13], [16]. We observed via immunofluorescence assay (IFA) that vFLIP localized predominantly to nuclear bodies in transfected 293T cells; however vFLIP expression was greatly reduced and difficult to detect at all by IFA in the presence of co-transfected RTA (Fig 2). This observation supported the hypothesis that RTA might be affecting vFLIP protein stability. To evaluate the role of RTA on vFLIP stability, 293T cells were transfected with myc-vFLIP in the presence and absence of RTA (Fig 3a). Co-transfection of RTA with vFLIP resulted in greatly decreased levels of vFLIP protein as detected by western blot (Fig 3a). This effect was dose dependent and inhibited by the proteasome inhibitor MG132 (Fig 3b and 3c) suggesting that RTA is inducing vFLIP degradation via the proteasome, rather than targeting vFLIP mRNA for degradation or inhibiting transcription.

### RTA inhibits vFLIP induced upregulation of the NFĸB responsive genes ICAM1 and TNFα

**Figure 4:**
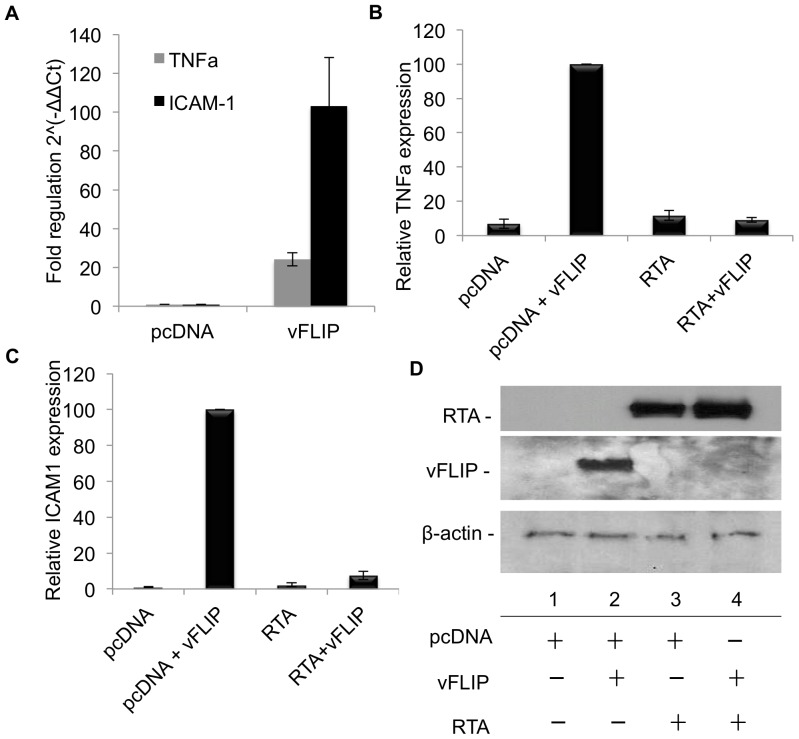
RTA inhibits vFLIP-induced expression of TNFα and ICAM1 in 293T cells. A-D. 293T cells were transfected with myc-vFLIP, RTA or empty vector control where indicated. At 72 hrs post-transfection, cells were harvested and split for RNA isolation and western blot (see D). For qPCR total RNA was isolated, reverse transcribed and quantified on an ABI7000 with using primers for TNFα and ICAM1 and Sybr green. The housekeeping genes used in the analysis were B-actin and GAPDH. Data was analyzed using the ΔΔCt method. A. vFLIP induced expression of TNFα and ICAM shown as fold regulation 2∧(-ΔΔCt). B and C. vFLIP induced expression of (B) TNFα, p = 0.0001 and (C) ICAM1, p = 0.0001 in the presence or absence of RTA calculated relative to vFLIP alone. Error bars represent standard error. D. Representative western blot from the same experiment, showing vFLIP and RTA protein expression, and RTA induced degradation of vFLIP.

To further examine the role of RTA on vFLIP function, we examined the affect of RTA on vFLIP induction of NFĸB responsive genes. We used an NFκB signaling pathway qPCR Array to identify genes whose transcripts were up-regulated by vFLIP in 293T cells and detected a number of genes that had been previously reported (Table 1) [19]–[21]. We chose to validate vFLIP-induced expression of TNFα and ICAM1 mRNA in the presence and absence of RTA in transfected 293T cells. TNFα was particularly interesting because it likely provides a positive feedback mechanism for maintaining latency since TNFα is a well characterized activator of NFĸB [22]. While both ICAM1 and TNFα expression were negligible in the empty vector transfected control cells, transfection of myc-vFLIP resulted in a 24 and 103-fold increases in TNFα and ICAM1 expression respectively (Fig. 4a). However, co-transfection of RTA with myc-vFLIP resulted in degradation of vFLIP (Fig. 4d) and an 11 and 13-fold reduction in TNFαa (Fig. 4b) and ICAM1 (Fig. 4c) expression, respectively, bringing gene expression down to background levels. RTA alone had no effect on TNFα and ICAM1 transcript levels (Figs. 4b and c).

**Table 1:** vFLIP induces expression of NFκB associated genes.

Description	RefSeq	Symbol	Fold regulation
Nuclear factor of kappa light polypeptide gene enhancer in B-cells 1	NM_003998	NFKB1	339.35
Activating transcription factor 1	NM_005171	ATF1	161.64
Signal transducer and activator of transcription 1, 91 kDa	NM_007315	STAT1	108.88
Tumor necrosis factor	NM_000594	TNF	65.64
Heme oxygenase (decycling) 1	NM_002133	HMOX1	55.97
Interleukin 1 receptor, type I	NM_000877	IL1R1	24.02
Interleukin 6 (interferon, beta 2)	NM_000600	IL6	17.10
Intercellular adhesion molecule 1	NM_000201	ICAM1	14.09
Caspase 1, apoptosis-related cysteine peptidase	NM_033292	CASP1	11.60
Tumor necrosis factor, alpha-induced protein 3	NM_006290	TNFAIP3	6.80
V-rel reticuloendotheliosis viral oncogene homolog	NM_006509	RELB	4.1
Interleukin 1, beta	NM_000576	IL1B	3.37
Interleukin 8	NM_000584	IL8	3.06
Toll-like receptor 3	NM_003265	TLR3	2.94
Toll-like receptor 4	NM_138554	TLR4	2.90
Inhibitor of kappa light polypeptide gene enhancer in B-cells, kinase epsilon	NM_014002	IKBKE	2.86
Nuclear factor of kappa light polypeptide gene enhancer in B-cells inhibitor, alpha	NM_020529	NFKBIA	2.43

**Figure 5:**
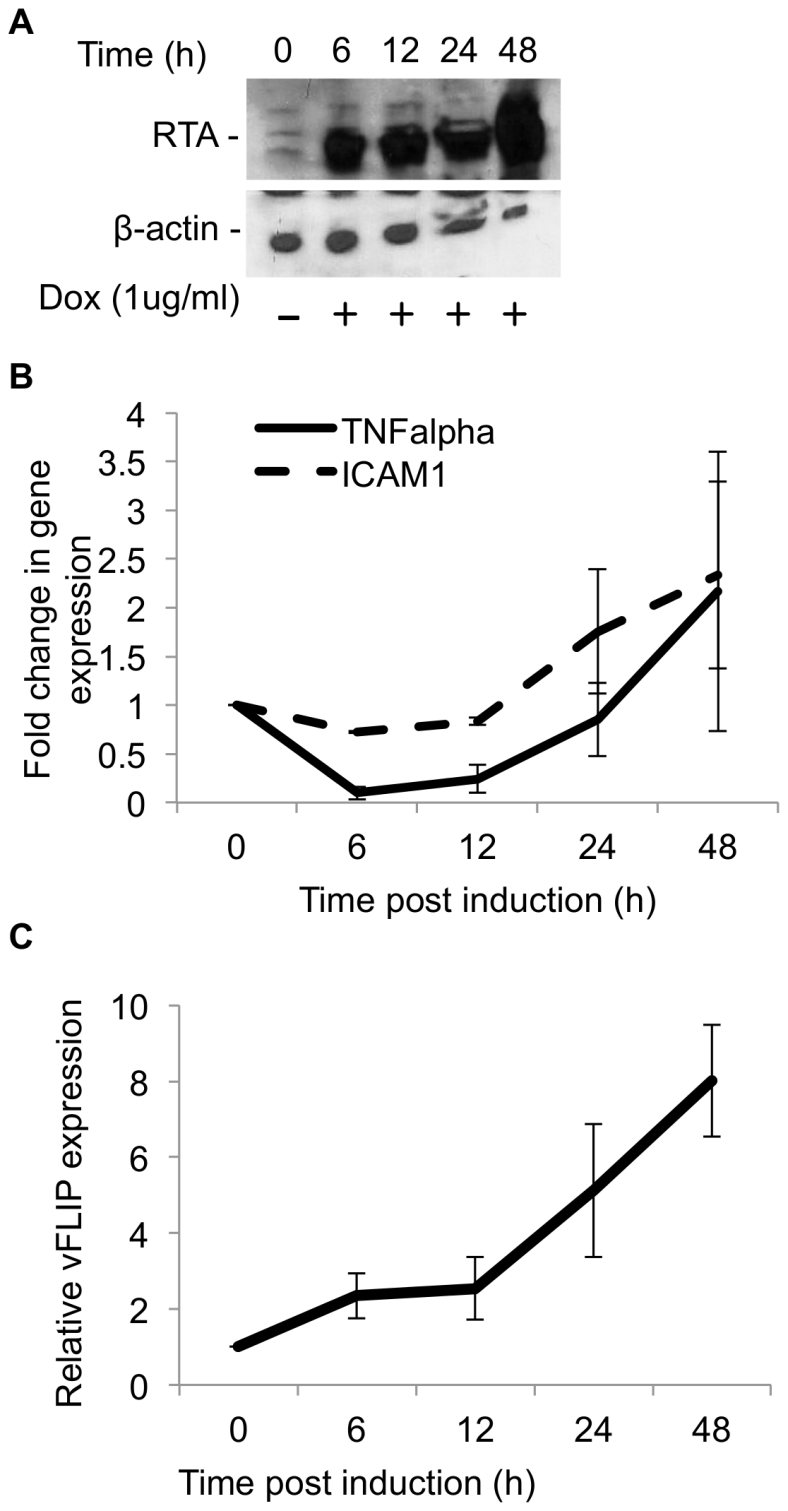
RTA inhibits vFLIP-induced expression of TNFα and ICAM1 in TREx BCBL1 RTA cells. A–C. TREx BCBL1 cells were induced with doxycycline (1 ug/ml) and harvested at the indicated time points. Cells were split for western to assess RTA induction and for RNA isolation for qPCR as described above. Results are expressed as fold regulation 2∧(-ΔΔCt) A. Cells harvested for protein were analyzed by SDS-PAGE followed by western blot against RTA and B-actin. B. qPCR analysis of TNFα and ICAM1 expression as explained above. C. qPCR analysis of vFLIP expression at the indicated time points post doxycycline induction. Error bars represent standard error.

We observed results in doxycycline induced TREx BCBL1 RTA cells that are consistent with those obtained in transfected 293T cells. Cells were treated with doxycycline for 0, 6,12, 24 and 48 hrs. RTA protein was detected after 6 hrs, indicating efficient lytic induction (Fig. 5a). Cells were analyzed at corresponding time points for TNFα and ICAM1 expression. Interestingly, both TNFα and ICAM1 RNA levels experienced a significant drop at 6 hrs followed by a rebound by 24 hrs (Fig. 5b). This data suggests that vFLIP mediated NFkB activity is transiently inhibited after RTA induction. This is consistent with previous reports of vFLIP expression during lytic replication and NFĸB activation later in lytic replication [12], [23]–[25].

To exclude the possibility that RTA was inhibiting vFLIP at the transcriptional level rather than at a post-translational level, we examined vFLIP transcript levels in doxycycline induced TREx BCBL1 RTA cells. As was previously reported, vFLIP mRNA was sufficiently expressed in TREx BCBL1 RTA cells (average ΔCT in uninduced cells was 5.9±0.037) and expression actually increases upon lytic induction, suggesting that RTA protein expression is not inhibiting vFLIP transcription [12], [23], [24], [26] (Fig. 5c).

### RTA induces vFLIP degradation through recruitment of a novel cellular ubiquitin ligase

**Figure 6:**
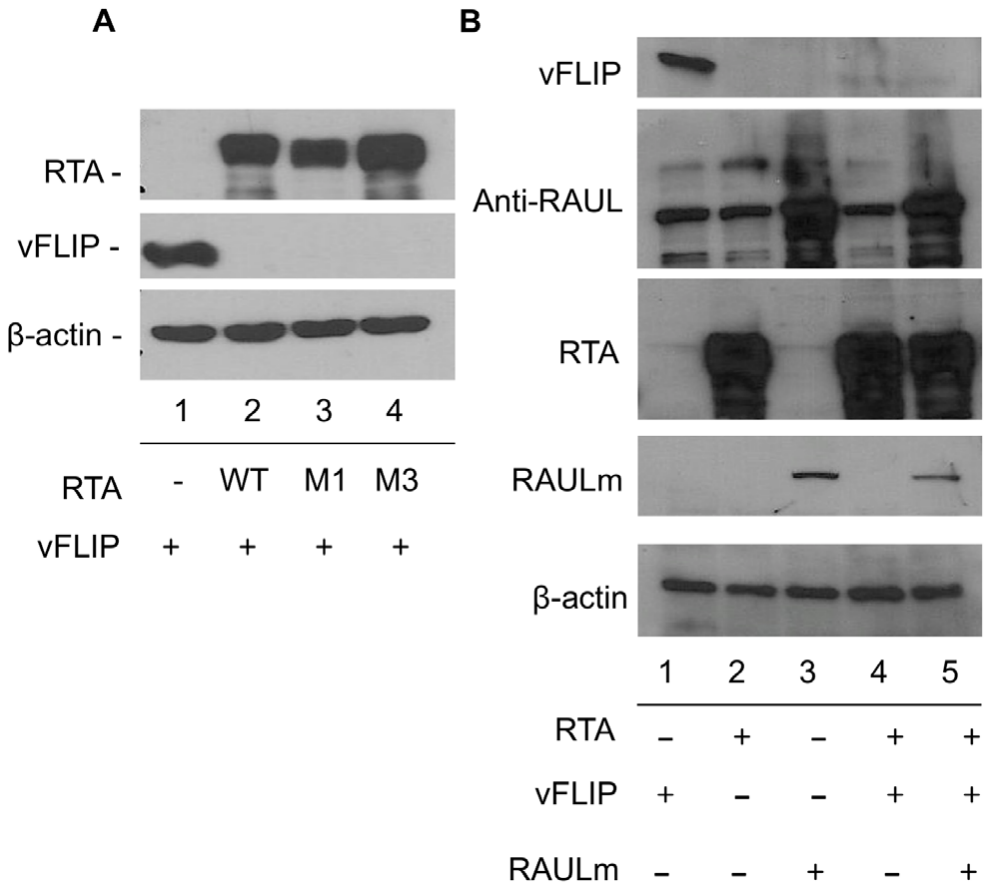
The ubiquitin ligase activities of RTA and RAUL are not required for RTA induced degradation of vFLIP. A. 293T cells were transfected with myc-vFLIP and wild type or mutant (M1-C141S and M3-H145L) RTA or control vector where indicated. 72 hrs post transfection, cells were harvested and analyzed by SDS-PAGE followed by western blot with antibody against RTA, myc(vFLIP) and B-actin. B. 293T cells were transfected with myc-vFLIP, RTA, and myc-RAUL mutant C1051A where indicated. 72 hrs post transfection, cells were harvested and analyzed by SDS-PAGE followed by western blot with antibody against myc(vFLIP), RAUL, RTA, myc(RAUL) mutant and B-actin.

RTA has been reported to participate both directly and indirectly in the degradation of several inhibitors of lytic replication [13]–[16]. To evaluate whether RTA is directly promoting the degradation of vFLIP, we employed two mutants in the ubiquitin E3-ligase domain of RTA, M1 and M3, in which C141 has been mutated to S and H145 to L respectively. These mutants cannot degrade IRF7 and M3 is also defective for degradation of Hey-1 and K-RBP [13]–[15]. However, in 293T cells co-transfected with vFLIP and either RTA mutant (M1 or M3), vFLIP protein levels were just as efficiently reduced as with wild-type RTA (Fig. 6a, lanes 2, 3 and 4), suggesting that while RTA is promoting vFLIP degradation, the degradation does not require the intrinsic ubiquitin E3-ligase activity of RTA. As well as having intrinsic E3 ubiquitin ligase activity targeting IRF7, RTA was previously shown to also recruit and stabilize the cellular HECT-ubiquitin E3-ligase RAUL leading to degradation of both cellular IRF3 and IRF7 [16]. To evaluate the possible role of RAUL in RTA induced degradation of vFLIP, we employed a dominant negative mutant of RAUL that has a C1051A mutation at the catalytic site in its HECT domain [17]. In the presence of RTA and endogenous RAUL, vFLIP is efficiently degraded (Fig. 6b, lane 4), however upon addition of the mutant myc-tagged RAUL ubiquitin E3 ligase in the presence of RTA, we did not observe any significant increase in vFLIP levels (Fig. 6b, lane 5). This data suggests that RTA-induced vFLIP degradation does not occur via a RAUL-dependent mechanism.

### Amino acids 11–150 in RTA are required for vFLIP degradation

**Figure 7:**
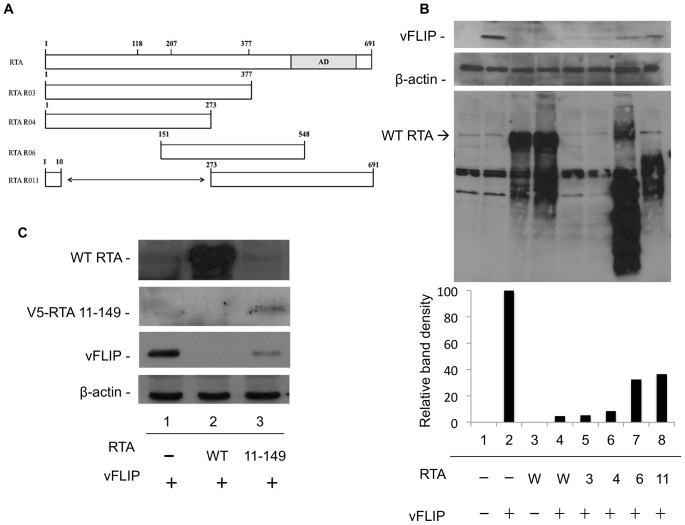
Amino acids 11–149 in RTA are required for degradation of vFLIP. A. Illustration depicting the RTA truncation mutants used in this study. Note that the rabbit RTA antibody recognizes amino acids 527–539, so not all mutants were detected by western blot. B. 293T cells were transfected with vFLIP and wild type or mutant RTA where indicated. 72 h post-transfection cells were harvested and analyzed by SDS-PAGE followed by western blot against myc(vFLIP), RTA and B-actin. C. 293T cells were transfected with V5 tagged RTA 11–149 and vFLIP where indicated. 72 h post-transfection cells were harvested and analyzed by SDS-PAGE followed by western blot against myc(vFLIP), RTA and B-actin.

Since RTA is not using its intrinsic ubiquitin ligase activity or RAUL to destabilize vFLIP, we hypothesized that RTA must be recruiting another cellular ubiquitin ligase. Therefore, to determine whether a specific domain of RTA is required for RTA induced degradation of vFLIP we used RTA truncation mutants to assess their effects on vFLIP stability. The results with RTA mutants R03(1–377), R04(1–273), R06(151–548), and R11(**Δ**11–272) are compared with wild-type RTA(1–691) in Fig. 7a. All of these mutants were previously shown to still bind to C/EBPαa and/or DNA, suggesting that despite their truncation they are still folding well enough to maintain some of their functional domains [18]. Co-transfection of R06 RTA(151–548) and R11(**Δ**11–272) with vFLIP resulted in a moderate defect in vFLIP degradation (Fig. 7b, compare lanes 4 to 7 and 8). The R03(1–377) and R04(1–273) proteins were not detected on western blots because they are missing the epitope recognized by our RTA antibody (located at amino acids 527–539). We feel confident, however, that expression of RTA was sufficient because vFLIP was completely degraded. RTA 11–149 retained the ability to induce vFLIP degradation, though not as efficiently as full length RTA (Fig. 7c). This data suggests that a region within amino acids 11–149 is likely to be critical for forming a stable interaction with either vFLIP or a cellular protein required for vFLIP destabilization or both. This region contains the ubiquitin ligase domain but is distinct from the region reported to bind IRF7.

## Discussion

It is well established that RTA activates immediate-early, early and late gene expression by transactivating multiple viral promoters. More recently RTA has been assigned intrinsic ubiquitin E3-ligase activity as well as been shown to interact with and stabilize the cellular ubiquitin E3-ligase RAUL. RTA has been shown to influence the stability of a number of cellular factors that may act as inhibitors of lytic reactivation including K-RBP, LANA, IRF7, IRF3 and Hey1. vFLIP is a latently expressed protein that promotes latency and has been reported to inhibit lytic reactivation in a cell type specific context through NFĸB activation and suppression of the AP1 pathway (27). NFκB activation has been shown to inhibit lytic reactivation and inhibition of NFκB signaling has been shown to promote lytic reactivation [11], [12], [27].

Our data suggests that RTA expression inhibits vFLIP induced NFκB activation, possibly as a mechanism to counteract promotion of latency by vFLIP, and may thus be necessary for either reactivation and/or the primary lytic infection process. RTA had no effect on TNFα induced NFκB activation suggesting that RTA is specifically targeting vFLIP, rather than components of the NFĸB pathway. We observed a dramatic decrease in vFLIP protein levels in the presence of RTA in co-transfection experiments. This effect was inhibited by treatment with MG132, suggesting that RTA is targeting vFLIP for degradation via the proteasome. RTA does not appear to affect vFLIP transcription, because vFLIP transcripts were shown to increase after doxycycline induction of RTA in TREx BCBL1-RTA cells.

Several RTA deletion mutants exhibited a defect in vFLIP degradation, supporting our postulation that RTA is promoting the degradation of vFLIP. We evaluated the possibility that degradation was occurring through the ubiquitin E3-ligase activity of RTA. We did not see any significant defect in proteolysis of vFLIP in the presence of the RTA ubiquitin ligase mutant. This suggests that RTA is recruiting a cellular protein to degrade vFLIP. This is a common strategy for viruses; they often degrade host proteins involved in the immune response. In fact, RTA has been shown to recruit and stabilize the cellular ubiquitin E3-ligase RAUL to target IRF3 and IRF7 for degradation leading to down-modulation of the interferon response [16]. We evaluated the role of RAUL in RTA induced degradation of vFLIP but observed no significant stabilization of vFLIP in the presence of a dominant negative RAUL ubiquitin E3-ligase mutant. This data suggests that RTA likely interacts with another ubiquitin ligase, possibly in the region of amino acids 11–150.

We also demonstrated that RTA inhibits expression of two vFLIP induced NFĸB responsive genes, TNFα and ICAM1, in both transfected 293T cells and TREx BCBL1-RTA cells, likely through degradation of vFLIP. This data provides further evidence for the role of RTA in inhibition of NFĸB early in lytic reactivation.

Overall, we have identified a novel previously unsuspected mechanism involved in activation of KSHV lytic replication in the presence of an established latent state. In addition to activating multiple viral lytic cycle gene promoters, the viral immediate-early regulatory proteins evidently must also need to remove viral and cellular blocks to or repressors of the induction of the lytic replication process. Specifically, we present evidence here that the KSHV lytic cycle trigger protein RTA abrogates latent state NFκB signaling by targeting the key KSHV-encoded latent state protein vFLIP for proteasomal degradation.

## References

[1] Ballestas ME (1999) Efficient Persistence of Extrachromosomal KSHV DNA Mediated by Latency-Associated Nuclear Antigen. Science 284: 641–644. 10213686 10.1126/science.284.5414.641

[2] Cotter M, Robertson E (1999) The latency-associated nuclear antigen tethers the Kaposi's sarcoma-associated herpesvirus genome to host chromosomes in body cavity-based lymphoma cells. Virology 264: 254–264. 10562490 10.1006/viro.1999.9999

[3] Sun R, Lin S-F, Gradoville L, Yuan Y, Zhu F, et al (1998) A viral gene that activates lytic cycle expression of Kaposi's sarcoma-associated herpesvirus. PNAS 95: 10866–10871. 9724796 10.1073/pnas.95.18.10866 PMC27987

[4] Chaudhary PM, Jasmin A, Eby MT, Hood L (1999) Modulation of the NF-kB pathway by virally encoded Death Effector Domains-containing proteins. Oncogene 18: 5738–5746. 10523854 10.1038/sj.onc.1202976

[5] Liu L, Eby MT, Rathore N, Sinha SK, Kumar A, et al (2002) The human herpes virus 8-encoded viral FLICE inhibitory protein physically associates with and persistently activates the Ikappa B kinase complex. J Biol Chem 277: 13745–13751. 11830587 10.1074/jbc.M110480200

[6] Field N, Low W, Daniels M, Howell S, Daviet L, et al (2003) KSHV vFLIP binds to IKK-gamma to activate IKK. J Cell Sci 116: 3721–3728. 12890756 10.1242/jcs.00691

[7] Matta H, Mazzacurati L, Schamus S, Yang T, Sun Q, et al (2007) Kaposi's sarcoma-associated herpesvirus (KSHV) oncoprotein K13 bypasses TRAFs and directly interacts with the IkappaB kinase complex to selectively activate NF-kappaB without JNK activation. J Biol Chem 282: 24858–24865. 17597077 10.1074/jbc.M700118200

[8] Sun Q, Matta H, Chaudhary PM (2003) The human herpes virus 8-encoded viral FLICE inhibitory protein protects against growth factor withdrawal-induced apoptosis via NF-kappa B activation. Blood 101: 1956–1961. 12406869 10.1182/blood-2002-07-2072

[9] Matta H, Chaudhary PM (2004) Activation of alternative NF-kappa B pathway by human herpes virus 8-encoded Fas-associated death domain-like IL-1 beta-converting enzyme inhibitory protein (vFLIP). PNAS 101: 9399–9404. 15190178 10.1073/pnas.0308016101 PMC438988

[10] Guasparri I, Keller S, Cesarman E (2004) KSHV vFLIP is essential for the survival of infected lymphoma cells. J Exp Med 199: 993–1003. 15067035 10.1084/jem.20031467 PMC2211879

[11] Brown HJ, Song MJ, Deng H, Wu T (2003) NF- κ B Inhibits Gammaherpesvirus Lytic Replication NF- B Inhibits Gammaherpesvirus Lytic Replication. J Virol 77: 8532–8540 10.1128/JVI.77.15.8532 12857922 PMC165238

[12] Grossmann C, Ganem D (2008) Effects of NFkappaB activation on KSHV latency and lytic reactivation are complex and context-dependent. Virology 375: 94–102. 18321555 10.1016/j.virol.2007.12.044 PMC2822626

[13] Yu Y, Wang SE, Hayward GS (2005) The KSHV immediate-early transcription factor RTA encodes ubiquitin E3 ligase activity that targets IRF7 for proteosome-mediated degradation. Immunity 22: 59–70. 15664159 10.1016/j.immuni.2004.11.011

[14] Yang Z, Yan Z, Wood C (2008) Kaposi's sarcoma-associated herpesvirus transactivator RTA promotes degradation of the repressors to regulate viral lytic replication. J Virol 82: 3590–3603. 18216089 10.1128/JVI.02229-07 PMC2268447

[15] Gould F, Harrison SM, Hewitt EW, Whitehouse A (2009) Kaposi's sarcoma-associated herpesvirus RTA promotes degradation of the Hey1 repressor protein through the ubiquitin proteasome pathway. J Virol 83: 6727–6738. 19369342 10.1128/JVI.00351-09 PMC2698570

[16] Yu Y, Hayward GS (2010) The ubiquitin E3 ligase RAUL negatively regulates type i interferon through ubiquitination of the transcription factors IRF7 and IRF3. Immunity 33: 863–877. 21167755 10.1016/j.immuni.2010.11.027 PMC3012379

[17] You J, Wang M, Aoki T, Tamura T, Pickart CM (2003) Proteolytic targeting of transcriptional regulator TIP120B by a HECT domain E3 ligase. J Biol Chem 278: 23369–23375. 12692129 10.1074/jbc.M212887200

[18] Wang SE, Wu FY, Fujimuro M, Zong J, Hayward SD, et al (2003) Role of CCAAT/Enhancer-Binding Protein Alpha (C/EBP α) in Activation of the Kaposi's Sarcoma-Associated Herpesvirus Protein (RAP) Promoter in Cooperation with the KSHV Replication and Transcription Activator (RTA) and RAP Role of CCAAT/Enhan. J Virol 77: 600–623 10.1128/JVI.77.1.600 12477864 PMC140597

[19] An J, Sun Y, Sun R, Rettig MB (2003) Kaposi's sarcoma-associated herpesvirus encoded vFLIP induces cellular IL-6 expression: the role of the NF-jB and JNK/AP1 pathways. Oncogene 22: 3371–3385 10.1038/sj.onc.126407 12776188

[20] Punj V, Matta H, Chaudhary PM (2012) A computational profiling of changes in gene expression and transcription factors induced by vFLIP K13 in primary effusion lymphoma. PloS One 7: e37498. 22624040 10.1371/journal.pone.0037498 PMC3356309

[21] Punj V, Matta H, Schamus S, Chaudhary PM (2009) Integrated microarray and multiplex cytokine analyses of Kaposi's Sarcoma Associated Herpesvirus viral FLICE Inhibitory Protein K13 affected genes and cytokines in human blood vascular endothelial cells. BMC Medical Genomics 2: 50. 19660139 10.1186/1755-8794-2-50 PMC2732924

[22] Rothe M, Sarma V, Dixit V, Goeddel D (1995) TRAF2-mediated activation of NF-kappa B by TNF receptor 2 and CD40. Science 269: 1424–1427. 7544915 10.1126/science.7544915

[23] Nakamura H, Lu M, Gwack Y, Zeichner SL, Jung JU, et al (2003) Global Changes in Kaposi's Sarcoma-Associated Virus Gene Expression Patterns following Expression of a Tetracycline-Inducible Rta Transactivator. J Virol 77: 4205–4220 10.1128/JVI.77.7.4205 12634378 PMC150665

[24] Sun R, Lin S, Staskus K, Grogan E, Haase A, et al (1999) Kinetics of Kaposi's Sarcoma-Associated Herpesvirus Gene Expression Kinetics of Kaposi's Sarcoma-Associated Herpesvirus Gene Expression. J Virol 73: 2232. 9971806 10.1128/jvi.73.3.2232-2242.1999 PMC104468

[25] Sgarbanti M, Arguello M, tenOever BR, Battistini A, Lin R, et al (2004) A requirement for NF-kappaB induction in the production of replication-competent HHV-8 virions. Oncogene 23: 5770–5780. 15235582 10.1038/sj.onc.1207707

[26] Krishnan HH, Naranatt PP, Marilyn S, Zeng L, Bloomer C, et al (2004) Concurrent Expression of Latent and a Limited Number of Lytic Genes with Immune Modulation and Antiapoptotic Function by Kaposi's Sarcoma-Associated Herpesvirus Early during Infection of Primary Endothelial and Fibroblast Cells and Subsequent Decline of. J Virol 78: 3601–3620 10.1128/JVI.78.7.3601 15016882 PMC371072

[27] Ye F-C, Zhou F-C, Xie J-P, Kang T, Greene W, et al (2008) Kaposi's sarcoma-associated herpesvirus latent gene vFLIP inhibits viral lytic replication through NF-kappaB-mediated suppression of the AP-1 pathway: a novel mechanism of virus control of latency. J Virol 82: 4235–4249. 18305042 10.1128/JVI.02370-07 PMC2293065

